# Retrograde Instillation of Methylene Blue in the Difficult Diagnosis of BPF

**DOI:** 10.1155/2012/714746

**Published:** 2012-10-04

**Authors:** F. Ravenna, C. Feo, N. Calia, C. Avoscan, C. Barbetta, G. N. Cavallesco

**Affiliations:** U. O. di Pneumologia, S. Anna Hospital, Ospedale S. Anna-Cona, 44122 Ferrara, Italy

## Abstract

We report two cases in which we were able to diagnose bronchopleural fistula through retrograde methylene blue instillation during bronchoscopy. In the first case, methylene blue was injected through an abdominal drain, followed by air instillation and detected in the left bronchial tree, demonstrating the presence of a fistula in the lingula's bronchus. In the second case, methylene blue was injected into a pleural drain, through a breach on a surgical suture and detected in the right bronchial tree, demonstrating the presence of a fistula in the right inferior bronchus. The retrograde instillation of methylene blue, through a drain in the abdomen or the thoracic wall, is a safe, cheap, and practical method that allows the bronchoscopist to identify the presence of a fistula and, more importantly, to identify the exact point on the bronchial tree where a fistula is located. This provides the possibility of sealing the fistula with a variety of devices. It is our opinion that this procedure should be considered a primary method of diagnosis when a bronchopleural fistula is suspected and a drain on the thoracic or abdominal wall is positioned such that effusions are able to drain.

## 1. Introduction

Bronchopleural fistulas (BPFs) are communications between the pleural space and the bronchial tree.They are an adverse complication of several pulmonary conditions, particularly in the postoperative period after lung surgery or other invasive lung procedures (such as bronchoscopy, lung/pleural biopsy) [1]. BPFs represent a challenge, both for their diagnosis and treatment. Pulmonary physicians are often consulted to assist in the management of these patients [6]. Several methods have been used to diagnose BPFs, including bronchography [2, 3], CT, and MRI [4, 5]. Bronchoscopy has been shown to play an important role in the diagnosis and treatment of these pathological conditions [6–8]. 

Bronchoscopy can be integrated with

- instillation of Xe 133 into the segmental bronchus leading to the fistula [9],
- instillation of other gasses such as ^81m^Kr and ^99m^Tc diethylene triamine pentaacetic acid ventilation scintigraphy [10],
- introduction of small metallic probes through the working channel of the bronchoscope, and changes in gas concentration in the pneumonectomy cavity after inhaling different concentrations of oxygen and N_2_O [11, 12],
- instillation of Indocianine green during bronchoscopy [13],
- antherograd instillation of methylene blue through the drain and its detection in the chest tube [14],
- retrograde instillation of methylene blue [15].

Methylene blue is an organic compound consisting of dark green crystals or crystalline powder, having a bronze-like luster. Solutions in water or alcohol have a deep blue color. Methylene blue is used as a bacteriologic stain and as an indicator. It inhibits guanylate cyclase and has been used to treat cyanide poisoning and to lower levels of methemoglobin [16]. It is a cheap and safe method of diagnosis, costing, on average, 4 euro for a 10 mL vial of 1% methylene blue. It has been used for many years in clinical practice, particularly during bronchoscopy [17], and can also be useful for the diagnosis of BPF. 

## 2. Case Presentation

We report two cases of BPF diagnosis achieved through retrograde instillation of methylene blue during bronchoscopy.

### 2.1. Clinical Case 1

G. B. male, 34 years old. 11 years ago the patient underwent a posttraumatic splenectomy. This was the result of 40 days experience of nausea and vomiting (food and biliary acids) for which he went to see his GP. The patient undergoes a thorax-abdomen CT scan (with contrast enhancement), which shows herniation of colon, peritoneum, stomach, and vessels trough the diaphragm. Admitted to General Surgery division, he undergoes an election hernioplasty procedure, complicated by haemoperitoneum. An urgent laparotomy is performed to stop the bleeding. In the postoperative period, amylase is detected from one of the abdominal stumps. After some days, the patient develops pneumonia with fever and intense cough. He performs a bronchoscopy, which shows biliary fluid in the right and left bronchial tree. Suspecting an empyema, Mr. G. B. undergoes a new CT scan which shows the presence of a loculated effusion. It extends from the retro-pancreatic subphrenic space to the base of the left lung, through a breach located on the surgical suture. Furthermore, cough is noted during the abdominal drains' washings. A new FBS is performed. During the procedure, methylene blue 5 cc is injected through the abdominal drain, followed by air instillation (Retrograde methylene Blue Injection). Methylene blue appears then in the left bronchial tree, demonstrating the presence of a fistula located in the lingula's bronchus associated with a pleural-peritoneal fistula. The pleural-peritoneal fistula could have played a role in the development of a BPF. The pneumonia is successfully treated with antibiotics. Considering the improvement of clinical conditions in the days following the procedure, the patient is discharged and followed up (conservative treatment). He is now well and has no symptoms.

### 2.2. Clinical Case 2

R. R. male, 77 years old, history of pulmonary TB and diabetes mellitus. On 2008 he undergoes right inferior lobectomy for a lung squamosus carcinoma (T1N1 M0-IA). On April 2009 a chest CT scan (with contrast enhancement) shows the presence of free air and “low density deposits of uncertain attribution” in the pleural space, leading to a suspected BPF. He is admitted to our Pneumology department for further investigation and a thoracic drain is positioned. During an FBS session, 2 cc of concentrated methylene blue are instilled through the work channel of the bronchoscope, in the right bronchial tree, close to the surgical suture. The aim is to show the presence of blue colorant in the boulau connected to the drain in the thoracic wall, however, the test fails. Considering the relative good health of the patient, he is discharged. Between September 2011 and November 2011, the patient begins to complain of a chronic cough; he is subsequently admitted to our Pneumology Department and undergoes two FBSs with Anterograd methylene blue injection (5 cc); however, the test fails to provide diagnosis as no methylene blue is detected in the pleural drain. On December 2011 there is still evidence of air and pleural effusion on chest radiography, so a new pleural drain is positioned. Suspecting an esophagus-pleural fistula, the patient undergoes a barium esophagram, but no fistula is detected. The only way for air to reach the pleural cavity is in our opinion through a BPF. In order to confirm our suspicions, we propose to Mr. RR a Bronchoscopy with Retrograde methylene blue Injection. During this final FBS session, a solution of 5 cc of methylene blue mixed in 50 cc of saline solution (NaCl 0.9%) is injected into the pleural drain. The colorant finally appears in the right bronchial tree, demonstrating the presence of a BPF, located in the right inferior bronchus, through a breach on the surgical suture. After the diagnosis of BPF, an FBS with rigid bronchoscope is then performed and histo-acryl glue (1 cc -BluGran) is used to close the fistula. One week later, the chest drain is removed and the patient is discharged.

## 3. Discussion

**Figure 1:**
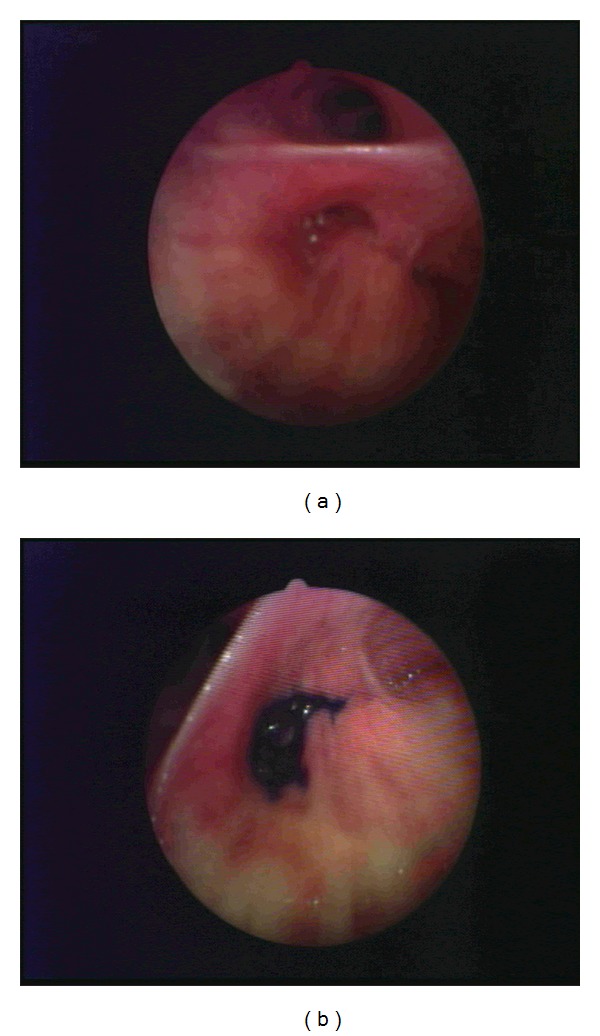
BPF before (a) and after (b) retrograde instillation of Methylene Blue.

Bronchopleural fistulas are an adverse complication related to various conditions (both surgical and nonsurgical). They have been reported after infections, gastroesophageal reflux disease with Barrett esophagus, malignancy, thoracic trauma, ARDS, necrotizing lung disease associated with radiation, and/or chemotherapy and could also be idiopathic. In lung cancer patients, BPFs are associated with an advanced stage, presence of residual tumor on the stump after surgery, and intrathoracic use of chemotherapy [1]. It is estimated that the incidence of BPFs range from 1.5 to 28% of cases following pulmonary resection and is highest in patients who undergo right pneumonectomy and right lower lobectomy. BPFs are often associated with empyema, a very severe complication of thoracic surgery and carry high rates of mortality and morbidity [18, 19]. Various complications can compromise survival, such as ARDS, aspiration pneumonia, respiratory dysfunction, infection, or destruction of the vascular stump [14]. BPFs are associated with multiple hospitalisations and comorbidities increasing costs for healthcare assistance. The diagnosis of BPF is not straightforward and presents a major challenge for pneumologists, radiologists, and other physicians involved with the presenting symptoms. An accurate diagnosis is important in order that the patient undergoes the proper treatment. This reduces the risk of infection and improves his quality of life. Several diagnostic methods have been shown to successfully diagnose Bronchopleural fistulas. The use of chest imaging has been reported as a successful method of diagnosis; however, there are also cases where CT and RMI have not proved to be a successful option. In addition, bronchoscopy does not consistently identify the fistula, particularly when it is small or when it is located out of reach of FBS. The use of gas instillation and ventilation scintigraphy with gases like Xe^133^, ^81m^Kr, or ^99m^Tc diethylene triamine pentaacetic acid can be a useful method of BPF diagnosis; however, there is difficulty in detecting the exact point where BPF is located. In contrast, a combination of FBS and instillation of a liquid colorant is not only able to accurately diagnose a BPF, but also to indicate the exact point of the BPF location, allowing posttreatment success to be easily determined. An anterograde instillation of methylene blue can help the physician with the diagnosis of BPF but is unable to determine the exact location of a BPF. In contrast, the retrograde instillation of methylene blue, through the drain in the abdomen or the thoracic wall, allows the bronchoscopist to identify the exact point on the bronchial tree where the BPF is located. Once the leak is located, it can be sealed with several devices, like histo-acril glue, valves, stents, and other devices aiming to stop air and liquid leaks (see Figure 1).

## 4. Conclusions

Retrograde methylene blue instillation during bronchoscopy is a useful and practical method used to accurately diagnose bronchopleural fistulas. This methodology is able to determine the exact point where the fistula is located, allowing the bronchoscopist to treat it properly. It has been shown to be more accurate than chest imaging and antherograd methylene blue instillation, because it is able to diagnose small Bronchopleural fistulas. With this cheap and practical method, we were able to achieve a diagnosis that several colleagues, using other methods, were not able to reach. It is our opinion that this procedure should be considered a primary method of diagnosis when a bronchopleural fistula is suspected and a drain on the thoracic or abdominal wall is positioned such that effusions are able to drain.
